# Serum Epithelial Biomarkers and Oxidative Stress Indicators in Acute Bronchiolitis: Association with Disease Severity and Recurrent Wheezing

**DOI:** 10.3390/children13060768

**Published:** 2026-06-01

**Authors:** Yeşim Yiğit, Özge Yılmaz, Ece Onur, Yurda Şimşek, Arzu Oran, Esra Toprak Kanık, Hasan Yüksel

**Affiliations:** 1 Department of Pediatric Hematology and Oncology, Balıkesir Atatürk City Hospital, Balıkesir 10100, Türkiye; 2 Department of Pediatric Allergy and Immunology, Faculty of Medicine, Celal Bayar University, Manisa 45030, Türkiye; 3 Department of Biochemistry, Faculty of Medicine, Celal Bayar University, Manisa 45030, Türkiye; 4 Department of Pediatric Allergy and Immunology and Pediatric Pulmonology, Faculty of Medicine, Celal Bayar University, Manisa 45030, Türkiye

**Keywords:** acute bronchiolitis, surfactant protein D, 8-isoprostane, CC16, YKL-40

## Abstract

**Highlights:**

**What are the main findings?**

- Serum YKL-40 levels were significantly associated with bronchiolitis severity.
- None of the evaluated biomarkers predicted recurrent wheezing at one-year follow-up.

**What are the implications of the main findings?**

- Acute-phase biomarkers may reflect disease severity but are insufficient for long-term prediction.
- Multifactorial mechanisms, including environmental and genetic factors, likely influence wheezing recurrence.

**Abstract:**

**Background:** Acute bronchiolitis is one of the most common lower respiratory tract infections in early childhood and is frequently associated with recurrent wheezing and later development of asthma. Identifying biomarkers related to airway epithelial injury and disease severity may improve risk stratification. **Materials and Methods:** A total of 155 children aged 1–36 months who presented with their first episode of wheezing were enrolled. Clinical data and bronchiolitis symptom scores were recorded at admission. Serum levels of CC16, surfactant protein-D (SP-D), YKL-40, and isoprostane were measured. Patients were followed for one year to assess recurrence of wheezing. **Results:** According to symptom scores, 81 patients had mild and 74 had moderate bronchiolitis; no severe cases were observed. The distribution of bronchiolitis severity differed significantly between recurrent and non-recurrent wheezing groups. Serum YKL-40 levels were significantly correlated with disease severity (*p* < 0.05), and the effect size analysis indicated a moderate effect. SP-D levels showed a non-significant trend with severity (*p* = 0.17). No significant associations were observed for CC16 or isoprostane. **Conclusions:** Serum YKL-40 may be a potential biomarker reflecting disease severity in children with acute bronchiolitis. Further longitudinal studies are needed to evaluate the prognostic value of epithelial injury markers for recurrent wheezing and asthma development.

## 1. Introduction

Acute bronchiolitis is a significant cause of morbidity in the pediatric population due to its high prevalence and association with future asthma development [1]. However, not all children exposed to infectious agents develop lower respiratory tract infections. Host factors, including immunological, structural, and functional characteristics of the airway, influence susceptibility to infection and the clinical severity of bronchiolitis [2]. The severity of bronchiolitis varies based on the degree of inflammation, viral virulence, and the host immune response. The epithelial response is particularly important, as it represents the first point of contact for viral pathogens upon entry into the respiratory tract [3].

Acute bronchiolitis remains one of the leading causes of hospitalization in infants worldwide, and its clinical course is highly variable, highlighting the need for reliable biomarkers to predict disease severity and long-term respiratory outcomes. Given that many children under the age of two are at risk for acute bronchiolitis and considering that it is the leading cause of hospitalization in infants under one year of age and is associated with asthma, there is a critical need to develop novel treatment strategies. These strategies require a comprehensive understanding of disease pathogenesis and immunology [4].

Several serum proteins indicative of epithelial permeability have been associated with asthma-related morbidity. Clara Cell 16 (CC16), present in both the respiratory tract and serum, protects airway tissues from oxidative stress. Changes in CC16 levels may vary depending on the extent and nature of epithelial injury; levels may increase due to leakage into the circulation or decrease due to impaired epithelial integrity and reduced production [5]. YKL-40, also known as chitinase-like protein, is secreted by proliferating cells such as osteoblasts and tumor cells. It is also produced by airway macrophages and neutrophils, which play a central role in the inflammatory processes underlying bronchiolitis. Its levels increase in conditions characterized by inflammation, fibrosis, and metastasis, particularly during extracellular matrix remodeling [6]. Similarly, deficiency in surfactant proteins contributes to various pulmonary disorders. Surfactant protein D (SP-D), a component of the pulmonary surfactant system secreted by type II alveolar cells, maintains alveolar humidity, facilitates elastic recoil during expiration, and reduces surface tension, thereby stabilizing alveoli and preventing collapse. It also prevents the transudation of interstitial and capillary fluids into alveoli and enhances macrophage-mediated clearance of inhaled particles [7].

Since recurrent wheezing is a predictor and/or risk factor for asthma in infancy, and as persistent inflammation is linked to airway remodeling, the role of mediators associated with remodeling and viral virulence becomes essential in predicting prognosis and recurrence [1].

Wheezing is a frequent symptom during childhood, and identifying recurrence risk is critical in early asthma diagnosis. Therefore, we aimed to investigate the potential roles of CC16, SP-D, isoprostane, and YKL-40 as biomarkers for recurrent wheezing in pre-school-aged children.

In addition to evaluating disease severity, we also aimed to investigate whether these biomarkers are associated with the risk of recurrent wheezing.

## 2. Materials and Methods

Bronchiolitis was defined as the first episode of wheezing associated with viral lower respiratory tract infection in young children, in accordance with established clinical guidelines. Patients with previous wheezing episodes were excluded to ensure accurate differentiation between bronchiolitis and recurrent wheezing phenotypes.

Inclusion criteria comprised children aged 1–36 months presenting with their first episode of wheezing. Exclusion criteria included a history of previous wheezing, chronic lung disease, congenital heart disease, immunodeficiency, and other chronic systemic illnesses.

A total of 155 patients meeting these criteria were included in the study. All patients were followed for 12 months after the initial episode. Recurrent wheezing was defined as the occurrence of two or more wheezing episodes during the follow-up period and was assessed prospectively. To avoid conceptual overlap, recurrent wheezing was analyzed separately from the initial bronchiolitis episode. Based on follow-up outcomes, patients were categorized into recurrent and non-recurrent wheezing groups. Follow-up data were obtained from outpatient visits and hospital records, and recurrent wheezing episodes were confirmed based on physician diagnosis.

Disease severity was assessed using a bronchiolitis symptom score, which was recorded for each participant at admission.

Written informed consent was obtained from all parents or legal guardians, and the study was approved by the local ethics committee (protocol no: 2013-118). Patient enrollment and sample collection were performed after obtaining ethics committee approval.

Demographic data including age, sex, past medical history (infections and allergies), parental smoking, daycare attendance, and family history were documented.

Nasal swabs were collected from 85 patients within the first three days of illness for respiratory viral panel analysis. Multiplex polymerase chain reaction (multiplex-PCR) was used to detect respiratory viral pathogens, including respiratory syncytial virus (RSV), rhinovirus, human metapneumovirus, coronavirus, adenovirus, influenza, parainfluenza, and bocavirus. Detailed information regarding the assay kit manufacturer and analytical platform used for multiplex-PCR analysis was unavailable due to the retrospective design of the study. Respiratory viral panel analysis was performed in 85 patients; however, complete viral data were available for 78 patients, who were included in the final subgroup analysis. The excluded cases were due to incomplete clinical data or insufficient sample quality for viral analysis.

Venous blood samples were obtained within the initial three days of acute illness, centrifuged, and stored at −80 °C for later analysis of SP-D, CC16, YKL-40, and isoprostane. Detailed information regarding the serum collection tube type and manufacturer was unavailable due to the retrospective nature of the study.

Serum SP-D levels were measured using ELISA kits from BioVendor Research Diagnostics (Brno, Czech Republic). YKL-40 levels were measured using ELISA kits from Quidel Corporation (San Diego, CA, USA), and 8-isoprostane levels were assessed using ELISA kits from Cayman Chemical (Ann Arbor, MI, USA). All biomarker measurements were performed in duplicate according to the manufacturers’ instructions. Optical density values were measured using a microplate reader, and final concentrations were calculated from standard calibration curves generated for each assay. Mean values of duplicate measurements were used for statistical analysis.

Symptom severity was evaluated using the scoring system proposed by Gajdos et al. Scores of 1–3 indicated mild disease, 4–8 moderate, and 9–12 severe disease [8]. Table 1 shows the bronchiolitis clinical severity scoring system used in this study.

Statistical analysis was conducted using SPSS v15.0 (Chicago, IL, USA), with a *p*-value < 0.05 considered statistically significant. Categorical variables were analyzed using Chi-square or Fisher’s exact test, while continuous variables were assessed using *t*-test and one-way ANOVA. Normality of data distribution was assessed using the Shapiro–Wilk test. Data were approximately normally distributed; therefore, parametric tests were applied. Given the exploratory nature of the study, no correction for multiple comparisons was performed. Multivariable logistic regression analysis was not performed due to the sample size and exploratory nature of the study.

Although bronchiolitis is most commonly defined in children under 2 years of age, patients up to 3 years were included if they presented with a first episode of wheezing and clinical findings consistent with acute bronchiolitis.

## 3. Results

### 3.1. Patient Characteristics

A total of 155 patients with a first episode of acute wheezing were enrolled. The mean age was 11.2 ± 8.9 months in the recurrent wheezing group and 10.9 ± 8.1 months in the non-recurrent group. Of the total patients, 94 (60.6%) were male. Among patients with recurrent wheezing, 43 (68.3%) were male. The demographic characteristics of patients with and without recurrent wheezing are presented in Table 2.

### 3.2. Bronchiolitis Severity and Recurrent Wheezing

Based on the bronchiolitis symptom score, 81 patients (52%) were classified as mild and 74 (48%) as moderate; no patients were categorized as severe. The distribution of bronchiolitis severity differed significantly between the recurrent and non-recurrent wheezing groups (*p* = 0.001).

A family history of asthma was associated with recurrent wheezing, although it was not associated with bronchiolitis severity. Similarly, a family history of transient wheezing was more prevalent in the recurrent group (*p* = 0.02).

Only three patients (1%) had a family history of drug or food allergy. One of these patients, who also had a family history of asthma and allergic rhinoconjunctivitis, experienced recurrence. However, no significant association was found between drug or food allergy history and bronchiolitis recurrence.

Children exposed to parental smoking tended to have more moderate bronchiolitis, although this association did not reach statistical significance (*p* = 0.07; Table 3). Recurrence risk was higher in children with siblings (49% vs. 23.5%), but this did not significantly correlate with bronchiolitis severity.

### 3.3. Respiratory Viral Panel Findings

Respiratory viral panel analysis was conducted in 85 patients; however, complete data were available for 78 patients included in the final analysis. The excluded cases were due to incomplete clinical data or insufficient sample quality. Recurrence rates were 33.3% in patients with no viral detection, 54.1% in those with a single viral agent, and 87.5% in those with multiple agents, based on subgroup analyses. This difference was statistically significant and confirmed by Fisher’s exact test. The relationship between serum biomarkers and viral parameters is presented in Table 4.

No statistically significant association was observed between bronchiolitis severity and viral detection status.

### 3.4. Serum Biomarker Analyses

Serum CC16 levels ranged from 2.89 to 21.89 ng/mL and were slightly higher in boys (12.1 ± 4.4) than in girls (11.9 ± 4.6). No statistically significant gender-based differences were noted for SP-D, YKL-40, CC16, or isoprostane levels. These markers were not evaluated during healthy periods; therefore, baseline comparisons were unavailable.

**Figure 1:**
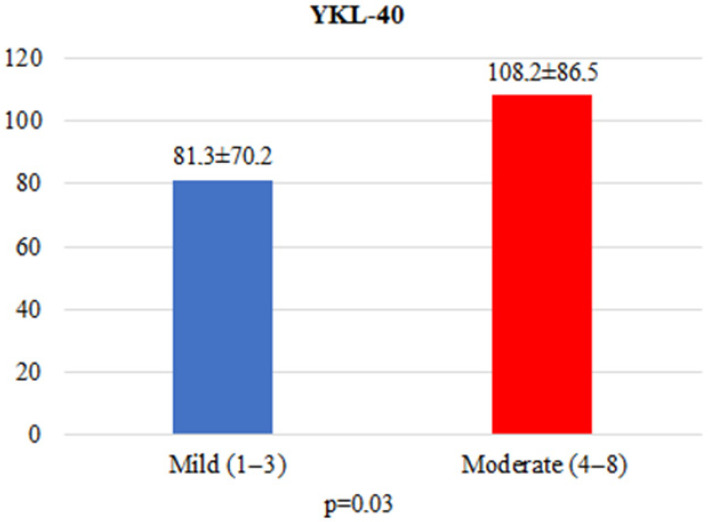
Comparison of serum YKL-40 levels according to bronchiolitis severity (mild vs. moderate). The x-axis represents bronchiolitis severity categories and the y-axis represents serum YKL-40 levels (ng/mL).

YKL-40 levels were 81.3 ± 70.2 in the mild group and 108.2 ± 86.5 in the moderate group. Bronchiolitis severity was significantly correlated with serum YKL-40 levels (*p* < 0.05), and the effect size (Cohen’s d) indicated a moderate effect. SP-D levels were inversely associated with disease severity but did not reach statistical significance (*p* = 0.17; Table 4). There were no significant associations for serum isoprostane (*p* = 0.5) or CC16 (*p* = 0.3; Table 5). Biomarker levels did not differ significantly based on the presence of viral pathogens (Table 6). Serum YKL-40 levels according to bronchiolitis severity are shown in Figure 1. Sensitivity analyses using non-parametric tests (Mann–Whitney U) yielded consistent results.

## 4. Discussion

In the present study, serum YKL-40 levels were significantly associated with bronchiolitis severity. Although SP-D levels showed a trend toward association, the relationship did not reach statistical significance. Acute bronchiolitis, particularly in children under 2 years of age, leads to epithelial damage and loss of barrier integrity in the airways, increasing the risk for chronic diseases like asthma [1,2,3,4,5]. Early identification of factors associated with disease severity and recurrent wheezing may improve clinical risk stratification and follow-up strategies.

Although CC16 levels were slightly higher in boys, previous research suggests that CC16 is not affected by gender, lipids, or BMI [9]. This minor gender difference may reflect the higher prevalence of bronchiolitis in boys. No association was found between secondhand smoke exposure and CC16 levels, potentially due to the timing of serum collection during the acute phase, rather than during health [10]. Since CC16 is filtered by the kidneys, children with chronic illnesses were excluded.

CC16 was not associated with recurrence or disease severity. As an indicator of acute lung injury [11], assessment during asymptomatic periods may provide different insights regarding baseline epithelial injury and airway inflammation. Further genetic studies may clarify CC16’s role, as it is encoded on chromosome 11.

YKL-40 levels were significantly associated with disease severity, but not with age, gender, or recurrent wheezing, consistent with previous studies [12]. The absence of an association between biomarkers and recurrent wheezing suggests that acute-phase inflammatory markers alone may not be sufficient to predict long-term respiratory outcomes, which are likely influenced by multiple environmental and genetic factors. In this context, YKL-40 has been proposed as a biomarker of airway remodeling and inflammation in pediatric asthma [13,14]. Our findings support the role of YKL-40 as a marker of acute airway inflammation severity during bronchiolitis rather than long-term susceptibility to recurrent wheezing. This finding further suggests that post-bronchiolitis wheezing may be influenced by factors beyond acute epithelial injury alone, including genetic predisposition, environmental exposures, and viral characteristics.

SP-D levels were higher in mild disease, aligning with previous findings in COPD and neonatal lung disease [15,16]. Although the results were not statistically significant, the trend warrants further research.

Isoprostanes are PG-like compounds formed via peroxidation of polyunsaturated fatty acids, primarily arachidonic acid [17]. In our study, isoprostane levels showed no significant relationship with severity or recurrence. Prior studies have produced inconsistent results, likely due to variable oxidative stress responses [18,19,20]. The absence of correlation with smoking may reflect alternative sources of oxidative stress in these patients.

Having a wheezing sibling was strongly associated with bronchiolitis recurrence. Although not predictive of disease severity, it may indicate an increased predisposition to airway inflammation. While most children in the study were experiencing their first wheezing episode, the presence of siblings with wheezing may still serve as an indirect risk factor for asthma development [21]. Furthermore, the absence of multivariable analysis limits the interpretation of independent predictors.

### Limitations

This study has several limitations. First, the absence of patients with severe bronchiolitis limits the generalizability of the findings, as the study population consisted only of mild and moderate cases. Second, biomarker levels were measured only during the acute phase of illness and not during healthy periods, preventing baseline comparisons. Third, the relatively small sample size and single-center design may further limit the generalizability of the results. Prematurity status was not separately analyzed and may have influenced bronchiolitis severity and recurrent wheezing outcomes. Differences in viral pathogens and possible co-infections may also have influenced biomarker levels and clinical severity. In addition, inclusion of patients up to 3 years of age may have introduced some heterogeneity in distinguishing bronchiolitis from early childhood wheezing phenotypes. Finally, the absence of multivariable analysis represents an important limitation of this study, as potential confounding factors could not be fully adjusted and independent predictors could not be identified. Future studies including severe bronchiolitis cases, longitudinal biomarker assessments, and multivariable analyses are warranted.

## 5. Conclusions

Serum YKL-40 may represent a potential biomarker reflecting airway inflammation and disease severity in children with acute bronchiolitis. Although SP-D showed a trend toward association, the relationship did not reach statistical significance. Further longitudinal studies are necessary to evaluate their role in predicting disease recurrence and progression to asthma. These findings may contribute to improved clinical risk stratification and follow-up strategies in children with acute bronchiolitis.

## Figures and Tables

**Table 1:** Clinical scoring in acute bronchiolitis.

Score	0	1	2	3
**Respiratory Rate (RR)**				
**0–2 months**		≤60	61–69	≥70
**2–12 months**		≤50	51–59	≥60
**12–24 months**		≤40	41–44	≥45
**Retractions**	None	Intercostal only	Intercostal/subcostal and substernal	Intercostal/subcostal and nasal flaring
**Wheezing**	None	End of expiration	Throughout	Audible without a stethoscope
**General appearance**	Normal	Mild irritability	Irritable and feeding difficulty	Confusion and feeding difficulty

**Table 2:** Demographic characteristics of patients.

	Recurrent Wheezing *n* = (63)	Non-Recurrent Wheezing *n* = (92)	*p*
Age *	11.2 ± 8.9	10.9 ± 8.1	0.82
Male **	43 (68.3%)	51 (55.4%)	0.109
Female **	20 (31.7%)	41 (44.6%)	0.109

* Mean ± SD. ** N (%).

**Table 3:** Relationship between parental cigarette smoking and bronchiolitis severity.

	Mild (1–3)	Moderate (4–8)	*p*
No parental smoking	53 (58.2%)	38 (41.8%)	0.07
Parental smoking	28 (43.8%)	36 (56.2%)	

Values are presented as row percentages.

**Table 4:** Relationship between serum proteins and viral parameters.

	Agent Not Detected	Single Agent	Multi Agents	*p*
SP-D	179.4 ± 108.7	169.1 ± 99.8	164.4 ± 74.1	0.88
CC16	12.3 ± 4.0	12.3 ± 3.7	10.5 ± 4.3	0.47
YKL-40	77.2 ± 61.6	64.4 ± 51.7	52.4 ± 30.0	0.42
Isoprostane	38.9 ± 10.3	37.8 ± 10.5	32.4 ± 8.03	0.28

One-way ANOVA test was used; *p* < 0.05 was considered significant. Post hoc analysis was performed using Tukey’s test.

**Table 5:** Relationship of serum proteins and disease severity.

	SP-D	YKL-40	CC16	Isoprostane
Mild (1–3) *	188.3 ± 100.1	81.3 ± 70.2	12.3 ± 4.5	38.1 ± 11.9
Moderate (4–8) *	164.8 ± 113.9	108.2 ± 86.5	11.7 ± 4.5	36.8 ± 12.8
*p*-value **	0.17	0.03	0.36	0.5

* Mean ± SD. ** Student’s *t*-test.

**Table 6:** Relationship of serum proteins and disease recurrence.

	Recurrent Wheezing *	Non-Recurrent Wheezing *	*p* **
SP-D	158.0 ± 86.0	190.2 ± 118.2	0.06
YKL-40	90.7 ± 78.2	96.4 ± 80.4	0.66
CC16	11.3 ± 3.9	12.4 ± 4.8	0.143
Isoprostane	36.2 ± 10.8	38.4 ± 13.2	0.268

* Mean ± SD. ** Student’s *t*-test.

## Data Availability

The data presented in this study are not publicly available due to patient privacy and ethical restrictions related to pediatric clinical data. However, anonymized data are available from the corresponding author upon reasonable request.
